# What are the factors that cause emergency home visit in home medical care in Japan?

**DOI:** 10.1002/jgf2.389

**Published:** 2020-10-30

**Authors:** Kaku Kuroda, Taro Miura, Shota Kuroiwa, Moe Kuroda, Naoko Kobayashi, Keiichiro Kita

**Affiliations:** ^1^ Department of Family Medicine SUNY Upstate Medical University Syracuse NY USA; ^2^ Toyama Machinaka Clinic Toyama Japan; ^3^ Department of General Medicine University of Toyama Toyama Japan

## Abstract

**Background:**

In the home medical care setting, the factors causing emergency home visits (EHV) remain unclear. This study aimed to determine those factors and examine their relationship with EHV requests.

**Methods:**

This is a single‐center retrospective observational study from data obtained from a home medical care clinic. We assessed the association between frequency of EHV and age, gender, level of care‐needed, cancer, and medical device in use with using Poisson regression analysis.

**Results:**

A total of 608 EHV in 214 patients were analyzed. Common chief complaints were fever, death, and dyspnea. As factors that affect frequency of EHV because of fever, higher care‐needed level (RR: 3.35; 95% CI: 1.95‐5.74, *P* < .001), urinary catheter use (RR: 1.94; 95% CI: 1.22‐3.08, *P* = .005), and central venous port use (RR: 2.39; 95% CI: 1.44‐3.96, *P* = .001) showed significant correlation. Regarding EHV because of dyspnea, lung tumor (RR: 2.71; 95% CI: 1.26‐5.84, *P* = .011) and home oxygen use (RR: 3.96; 95% CI: 2.05‐7.68, *P* < .001) showed significant correlation. Regarding EHV because of all chief complaints, higher care‐needed level (RR: 1.59; 95% CI: 1.12‐2.26, *P* = .009), urinary catheter use (RR: 1.78; 95% CI: 1.13‐2.93, *P* = .014), and central venous port use (RR: 1.75; 95% CI: 1.04‐2.93, *P* = .034) showed positive correlation.

**Conclusion:**

The factors associated with frequency of EHV because of fever or all chief complaints were urinary catheter use, central venous port use, and higher care‐needed level. As for dyspnea, they were lung cancer and home oxygen use. Our study suggests that the burdens on medical staffs, patients, and their families can be reduced through recognizing these risk factors.

## Graphical abstract

We identified the factors that affect frequency of emergency home visit (EHV) at home medical care support clinics by analyzing the reason for EHV and background factors observable in our clinic. Our study suggests that we can reduce the physical and psychology burden on medical staff, patients, and their families through recognizing these risk factors for EHV.

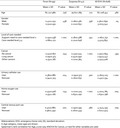

## 1 INTRODUCTION

While end‐of‐life care is attracting attention nowadays, it is reported that more than half of Japanese people desire to spend their time at home during their end of life.^1^ The Ministry of Health, Labour, and Welfare promotes a home medical care system for older adults to supply medical and nursing care in places where they are used to living.^2^ However, most people die in the hospital, and according to recent data, only about 10% die at home overall in Japan.^3^ To achieve end‐of‐life care at home, there is an urgent need to improve the home medical care system.

On the other hand, more than 70% of the physicians in home medical care support clinics feel burdened by the 24 hour on‐call system.^4^ Furthermore, sudden changes in a patient's condition which require an emergency home visit (EHV) become a burden for patients and their family.^5^ Moreover, it can become both a psychological burden and an obstacle to continue home medical care for home visiting nurses who have to request a physician's EHV.^6^ For enhancement of home medical care, it is essential to take measures to reduce physical and psychological burdens for patients and their family, physicians, and home visiting nurses. For that reason, we need to know about the details relating to EHV.

In a previous study, there is cross‐sectional work on contents of EHV,^7^ and it shows that the common reasons for EHV are fever, death, dyspnea, and cough in that order of prevalence. However, there is no direct study that investigates the factors that cause EHV so far and data are lacking.

This study aims to clarify the factors that cause EHV at home medical care support clinics by analyzing the reason for EHV and background factors observable in our clinic.

## 2 METHODS

### 2.1 Study design and participants

This descriptive cross‐sectional study was conducted by analyzing Toyama Machinaka Clinic's medical records related to EHV retrospectively. Toyama Machinaka Clinic is directly managed by Toyama city and is an enhanced home medical care support clinic with 3 physicians and 4 nurses. Toyama City has a population of approximately 417 000,^8^ and this clinic covers patients in all area of Toyama City. Most patients' demographics are complex, because of medical, social, and geographical reasons, which makes it difficult to be seen in local clinics.

We enrolled 214 patients over the age of 65 out of a total of 251 patients who had medical records within the 2 year duration from January 1, 2018, to December 31, 2019. In this study, patients aged 65 years or older were included because the population of patients under the age of 64 was small in our clinic, and therefore, this age‐group became nonrepresentative. We set the observation period as 2 years because the medical system of our clinic, which is in the fourth year since establishment, had been set up to some extent. We determined that a sufficient number of samples could be obtained after confirming that there is no significant change or bias in the number and presenting problems of our patients in the first and second years.

This study was conducted in accordance with “Ethical Guidelines for Medical and Health Research Involving Human Subjects” by the Ministry of Health, Labour, and Welfare and was approved by the ethics committee of Toyama University Hospital. (Approval number: R2019168).

We defined EHV as an examination which was performed by a visiting physician, requested by a patient, family member, caregiver, or the insurance medical institution, and contact was made mostly by phone when a physician acknowledges the need for an emergency visit to the patient's home.^9^

### 2.2 Statistical analysis

We analyzed a total of 608 EHV in 214 patients.

First, we conducted a descriptive analysis about chief complaints requiring EHV, time period, and background of patients. We defined chief complaints in this study as the reason why EHV was requested. We did not include the complaints that the patient stated or added when examined by the physician.

Second, we performed a Poisson regression analysis to determine the factors associated with the frequency of EHV. Variables include age, gender, level of care‐needed, presence or absence of cancer, and medical device in use (urinary catheter, home oxygen, and central venous port).

The level of statistical significance was set at *P* < .05. All statistical analyses were conducted using IBM SPSS Statistics version 26.

## 3 RESULTS

The number of participants was 214. Mean age ± standard deviation was 81.9 ± 8.4 years. The number of male and female patients was 96 and 118, respectively.

The total number of EHV was 608 (during normal working hours: 384; after‐hours: 224). Three common chief complaints that triggered the request for EHV were fever, death, and dyspnea. Among these, after‐hour visits were 39 for fever (17.4%), 56 for death (25.0%), and 12 for dyspnea (5.4%). We found that death was the most common complaint triggering the request of EHV in after‐hour visits.

Table 1 shows the results of the cross‐tabulation of each variable. This table shows the mean and standard deviation of the number of EHV for each chief complaint (column) for each subgroup of independent variables (row). Regarding the level of care‐needed, the care need criteria classified by the Japanese Long‐Term Care Insurance system was used to define the care need of individuals aged 65 or older into 7 levels: *support‐needed* levels 1 and 2, and *care‐needed* levels 1 to 5.^10^ These are categorized into two groups, *support‐needed* 1 and 2 and c*are‐needed* up to level 2 and *care‐needed* levels 3‐5 according to prior literature.^11^ The presence or absence of cancer was divided into three groups: no cancer, lung tumor (primary and metastatic lung cancer), and other cancers, because it was expected that a lung lesion in all cancers would act as a confounding factor, especially in EHV with the chief complaint of dyspnea.

**Table 1:** Cross‐tabulation of the mean number of EHV visits for each independent variable

	Fever (N = 99)		Dyspnea (N = 42)		All EHV (N = 608)	
	Mean ± SD	*P*‐value	Mean ± SD	*P*‐value	Mean ± SD	*P*‐value
Age	82.2 ± 7.980	.746	79.6 ± 7.289	.121	82.2 ± 8.341	.480
Gender						
Male	0.41 ± 1.032	.458	0.18 ± 0.481	.356	2.56 ± 2.690	.04
Female	0.51 ± 0.931		0.21 ± 0.652		3.07 ± 3.542	
Level of care‐needed						
Support need to care‐needed level 2	0.19 ± 0.538	<.001	0.18 ± 0.488	.352	2.06 ± 1.757	<.001
Care‐needed level 3‐5	0.68 ± 1.171		0.21 ± 0.648		3.47 ± 3.852	
Cancer						
No cancer	0.52 ± 0.877	.043	0.13 ± 0.524	<.001	2.50 ± 2.853	.393
Lung cancer	0.09 ± 0.373		0.54 ± 0.950		2.94 ± 3.447	
Other cancers	0.55 ± 1.187		0.12 ± 0.364		3.15 ± 3.406	
Urinary catheter use						
User	0.38 ± 0.887	.024	0.19 ± 0.573	.300	5.26 ± 5.125	<.001
Nonuser	0.94 ± 1.315		0.26 ± 0.631		2.43 ± 2.534	
Home oxygen use						
User	0.45 ± 0.918	.145	0.12 ± 0.415	<.001	4.10 ± 4.784	.004
Nonuser	0.55 ± 1.287		0.65 ± 1.050		2.63 ± 2.798	
Central venous port use						
User	0.40 ± 0.882	.002	0.19 ± 0.595	.580	4.74 ± 4.535	.005
Nonuser	0.96 ± 1.492		0.26 ± 0.449		2.61 ± 2.923	

Note

In Age category, mean ages are shown. Spearman's rank correlation for Age, a one‐way ANOVA for Cancer, a *t* test for other variables are used.

Abbreviations: EHV, emergency home visits; SD, standard deviation.

Spearman's rank correlation was used for “Age” category, a one‐way ANOVA was then conducted for “Cancer” category, and a *t* test was used for other variables. For EHV with the chief complaint of fever, significance was shown in the higher level of care‐needed, cancer, urinary catheter use, and central venous port use. For EHV with the chief complaint of dyspnea, it showed significance for lung cancer and home oxygen use. The evaluation for EHV for all chief complaints found significance in the higher level of care‐needed, urinary catheter use, home oxygen use, and central venous port use.

Finally, we performed a Poisson regression analysis for variables including age, gender, level of care‐needed, cancer, and medical device in use (urinary catheter, home oxygen, and central venous port). Tables 2, 3, 4 show the results of the analysis for the correlation between independent variables and relative risk (RR) of EHV for each chief complaint. For EHV because of all chief complaints, we conducted a negative binomial regression analysis because the mean was significantly larger than the variance. We checked VIF (Variable Inflation Factor) to consider collinearity between independent variables, and all VIFs were less than 5.000. Thus, we determined that there was no collinearity issue statistically.

**Table 2:** Poisson regression analysis for EHV because of fever

	RR	95% CI		*P*‐value
Age	1.00	0.98	1.03	.852
Gender				
Male	1.00 (ref)			
Female	1.15	0.76	1.74	.523
Level of care‐needed				
Support need to care‐needed level 2	1.00 (ref)			
Care‐needed level 3‐5	3.35	1.95	5.74	<.001
Cancer				
No cancer	1.00 (ref)			
Lung cancer	0.01	0.06	0.60	.005
Other cancers	1.02	0.65	1.62	.918
Urinary catheter use	1.94	1.22	3.08	.005
Home oxygen use	1.28	0.74	2.22	.384
Central venous port use	2.39	1.44	3.96	.001

Abbreviations: 95% CI, 95% confidence interval; EHV, emergency home visits; ref, reference; RR, relative risk.

**Table 3:** Poisson regression analysis for EHV because of dyspnea

	RR	95% CI		*P*‐value
Age	0.97	0.93	1.01	.090
Gender				
Male	1.00 (ref)			
Female	1.36	0.72	2.56	.342
Level of care‐needed				
Support need to care‐needed level 2	1.00 (ref)			
Care‐needed level 3‐5	1.19	0.61	2.29	.613
Cancer				
No cancer	1.00 (ref)			
Lung cancer	2.71	1.26	5.84	.011
Other cancers	0.87	0.35	2.13	.755
Urinary catheter use	1.10	0.48	2.53	.826
Home oxygen use	3.96	2.05	7.68	<.001
Central venous port use	1.16	0.47	2.85	.749

Abbreviations: 95% CI, 95% confidence interval; EHV, emergency home visits; ref, reference; RR, relative risk.

**Table 4:** Negative binomial regression analysis for EHV because of all chief complaints

	RR	95% CI		*P*‐value
Age	1.00	0.98	1.02	.835
Gender				
Male	1.00 (ref)			
Female	1.10	0.79	1.54	.574
Level of care‐needed				
Support need to care‐needed level 2	1.00 (ref)			
Care‐needed level 3‐5	1.59	1.12	2.26	.009
Cancer				
No cancer	1.00 (ref)			
Lung cancer	1.26	0.76	2.08	.372
Other cancers	1.14	0.77	1.69	.513
Urinary catheter use	1.78	1.13	2.93	.014
Home oxygen use	1.21	0.75	1.95	.428
Central venous port use	1.75	1.04	2.93	.034

Abbreviations: 95% CI, 95% confidence interval; EHV, emergency home visits; ref, reference; RR, relative risk.

We analyzed two common chief complaints including fever and dyspnea, which are avoidable, and EHV for all chief complaints. For factors that affect the occurrence of EHV because of fever, higher level of care‐needed (RR: 3.35; 95% CI: 1.95‐5.74, *P* < .001), urinary catheter use (RR: 1.94; 95% CI: 1.22‐3.08, *P* = .005), and central venous port use (RR: 2.39; 95% CI: 1.44‐3.96, *P* = .001) showed positive correlations. For EHV because of dyspnea, lung tumor (RR: 2.71; 95% CI: 1.26‐5.84, *P* = .011) and home oxygen use (RR: 3.96; 95% CI: 2.05‐7.68, *P* < .001) showed positive correlations. For EHV because of all chief complaints, the higher care‐needed level (RR: 1.59; 95% CI: 1.12‐2.26, *P* = .009), urinary catheter use (RR: 1.78; 95% CI: 1.13‐2.93, *P* = .014), and central venous port use (RR: 1.75; 95% CI: 1.04‐2.93, *P* = .034) showed positive correlations.

## 4 DISCUSSION

This study investigated factors that affect the frequency of EHV for cases at a home medical care support clinic. The results revealed the following three perspectives.

First, the most common chief complaint that caused the request for EHV was “fever”. And its related factors to note were urinary catheter use, central venous port use, and higher level of care‐needed. It has already been known that long‐term use of urinary catheters in admitted patients could be a bacteremia risk.^12^ However, this is the first report that mentions home care patients as far as we could discover. This result indicates that evidence‐based practices, such as reconsidering indications,^13^ regular replacement of indwelling catheter,^14^ and education regarding catheter care measures and hand‐washing compliance for medical staff,^15^ could reduce EHV for this complaint. Similarly, for central venous ports, risk factors for fever events include thrombosis and infection as complications for long‐term use, and poor performance status requires chemotherapy.^16^, ^17^ It is crucial to be aware of risks, explaining them to patient and family in advance, clean operation and education, careful examination to recognizes risks, and early detection of abnormal findings.^18^, ^19^ Higher level of care‐needed is also associated with fever events, as pointed out in previous studies.^11^, ^20^ Yokobayashi et al^20^ state the reason why level of care‐needed for fever events includes increased risk of aspiration because of less strength to cough, and increased susceptibility to infections from decreased muscle strength and poor nutrition status.

Second, dyspnea is a frequent chief complaint after fever, and an association to be noted was shown between EHV because of dyspnea and lung tumor/home oxygen use. Patients with lung tumors and home oxygen users should be considered a high‐risk group. Patients with lung cancer presenting with dyspnea are reported to have significantly shorter survival than patients with other cancers.^21^ Thus, it is definitely important to discuss with patients with lung cancer and their family about the possible occurrence of dyspnea in advance. By and large, it is known that high rates of emergency room visits in terminally ill patients and physician house calls are low‐quality indicators of end‐of‐life care.^22^, ^23^ However, most emergency room visits can be avoided by appropriate care and that can lead to improvement in patient satisfaction and better outcomes.^24^, ^25^ Although emergency room visits differ from EHV, we believe it is important to recognize that cancer is an associated factor for EHV in home medical care as well. The occasions that patients and their family feel the necessity to request EHV can be reduced by appropriate symptom management,^26^ increasing home care nursing,^27^ palliative care with team coordination,^28^ and education and support for family caregivers.^29^, ^30^

Fever and dyspnea are also valued symptoms in previous studies,^7^, ^11^, ^20^ and the results in this study correspond with those of previous studies.

Third, the higher care‐needed level, urinary catheter use, and central venous port use were extracted as factors associated with EHV because of all chief complaints. For these patients and their family, it is speculated that promoting understanding of the patient's disease and preparing for expected events can lead to quality improvement of EHV.

Two limitations of this study should be acknowledged. First, this study was a retrospective, single‐center study. However, we believe this study ensures universality to some extent because the results are consistent with previous studies and the use of 2 year‐round samples to prevent analysis of a clustered cohort. To increase external validity more, further studies including a prospective study with a longer duration and subgroup analysis for cases that requested EHV frequently are needed. Second, this study is an analysis of the factors that affect the frequency of EHV, not an analysis of diagnosis of the symptom which causes EHV. In general, the accurate diagnosis of cause of fever in elderly patients in a facility outside of hospital is difficult.^31^ Based on the current situation above, we assessed the problem of patients who needed EHV from the perspective of patient care.

In conclusion, common chief complaints that triggered the request of EHV were fever, death, and dyspnea. For factors that affect the occurrence of EHV because of fever, urinary catheter use, central venous port, and a higher level of care‐needed showed significant positive correlation. For EHV because of dyspnea, lung tumor and home oxygen use showed significant correlations. Regarding EHV because of all chief complaints, the higher care‐needed level, urinary catheter use, and central venous port use showed positive correlations. Our study suggests that we can reduce the physical and psychology burden on medical staffs, patients, and their families through recognizing these risk factors for EHV.

## CONFLICT OF INTEREST

Authors declare no conflict of interests in this article.
